# Protection of cholinergic and antioxidant system contributes to the effect of Vitamin D_3_ ameliorating memory dysfunction in sporadic dementia of Alzheimer’s type

**DOI:** 10.1080/13510002.2019.1617514

**Published:** 2019-05-17

**Authors:** Marilia Valvassori Rodrigues, Jessié Martins Gutierres, Fabiano Carvalho, Thauan Faccin Lopes, Vitor Antunes, Pauline da Costa, Maria Estér Pereira, Maria Rosa Chitolina Schetinger, Vera M. Morsch, Cinthia Melazzo de Andrade

**Affiliations:** a Departamento de Química, Centro de Ciências Naturais e Exatas, Universidade Federal de Santa Maria, Santa Maria, Brazil; b Departamento de Pequenos animais, Hospital Vetrinário, Universidade Federal de Santa Maria, Santa Maria, Brazil

**Keywords:** Acetylcholinesterase, dementia, vitamin D_3_, oxidative stress, rats, behavioral tests

## Abstract

**Objective:** Investigate Vitamin D_3_ (VD_3_) effect on the Acetylcholinesterase (AChE), oxidative damage and behavioral tests in animals subjected to Intracerebroventicular injection of Streptozotocin (ICV-STZ) simulating a Sporadic Dementia of Alzheimer's Type (SDAT) and treated with VD_3_ (21 days).

**Methods:** Animals were divided into eight groups: Vehicle, VD12.5 μg/kg, VD42 μg/kg, VD125 μg/kg, STZ, STZ+VD12.5 μg/kg, STZ+VD42 μg/kg, STZ+VD125 μg/kg.

**Results:** VD_3_ prevented the increase in AChE in groups of VD42 µg/kg and VD125 µg/kg; in AChE of synaptossomes and TBARS levels prevented the increase in group VD125 µg/kg; in ROS levels there was not a significant difference; for the Carbonyl Content all doses prevented the increase. Total Thiols prevent the decrease in VD42 µg/kg and VD125 µg/kg, and Reduced Glutathione prevented the decrease in VD125 µg/kg, Oxidized Glutathione prevented the increase in VD125 µg/kg. In relation to behavioral tests, the VD_3_ prevented the increase in time to find (days 2 and 3), in the time to find the platform (day 3) and in time spent in the quadrant (day 2). However, in relation to crossings there was not difference in groups. These results indicated the therapeutic effect of the VD_3_ in model of STZ in rats.

## Introduction

Dementia is a brain disorder characterized by a decline in mental functions, causing impairments in daily functions [1]. It affects over 20 million individuals worldwide [2,3] and its incidence has increased over the years [3,4].

In our previous works, we used intracerebroventricular injection of Streptozotocin (ICV-STZ) as a model of Sporadic Dementia of Alzheimer's type (SDAT) [5], for a well-established model, since it simulates pathological processes of Alzheimer Disease (AD), impaired brain glucose and energy, [6] worsening metabolic and cholinergic functions in the cerebral cortex of rats [5], besides increasing the oxidative stress [7].

Studies have shown that the impairment in the cholinergic function is of critical importance in the AD especially the brain areas of learning, memory and emotional responses, such as cortex cerebral [5,8].

Acetylcholinesterase (AChE) is an important regulatory enzyme that is found mainly in muscles and cholinergic neurons [9]. It is sensitive to reactive oxygen species production (eROS) [5,8]. The free radicals and oxidative stress have been shown as causes of the behavioral impairments and memory deficits in neurodegenerative disorders, such as AD [10]. Furthermore, the eROS in cells may be the cause of lipid peroxidation (LPO), which destroys the integrity of the cell membrane, breaking organelles or cells, causing thus the cell death [11,12].

In our organism, there are antioxidant defenses, which are important because they remove the free radicals, providing protection for biological sites [13] such as the Glutathione Oxidized (GSSG), Glutathione Reduced (GSH), Total Thiols, Carnobyl protein and Vitamin C.

There is evidence that the Vitamin D_3_ (VD_3_) has influence in age-related conditions and dementia [14,15]. So, it has been receiving attention. The VD_3_ may be involved in neuroprotective function [16,17] regulating neurotransmission, neuroprotection, brain processes and neuroimmunomodulation [18,19], as well as protecting against neurodegeneration in models of AD [16,20]. VD_3_ acts through its receptors (VDR) distributed by the brain [21], important area for cognitive processes [22]. In addition, VD_3_ is able to reduce the oxidative stress since it can prevent reactive oxygen species (ROS), protecting the cells from death [23], increasing the key anti-oxidative levels [24,25]. Thus, this study was undertaken in order to evaluate the effects of VD_3_ in the AChE, in oxidative stress markers and in behavioral parameters in rats with SDAT.

### Animals

A total of 56 male Wistar rats (90 days old; 350–400 g) were used for the assays for the behavioral test and AChE activity in synaptosomes was measured in 40 male rats (same age/weight); they were kept in the Central Animal House of Federal University of Santa Maria in regulated ambient temperature with free access to food and water. The ethical committee of UFSM (23081.003601/2013–21) approved all the procedures.

### Intracerebroventricular (ICV) injection of streptozotocin (STZ)

**Figure 1.**
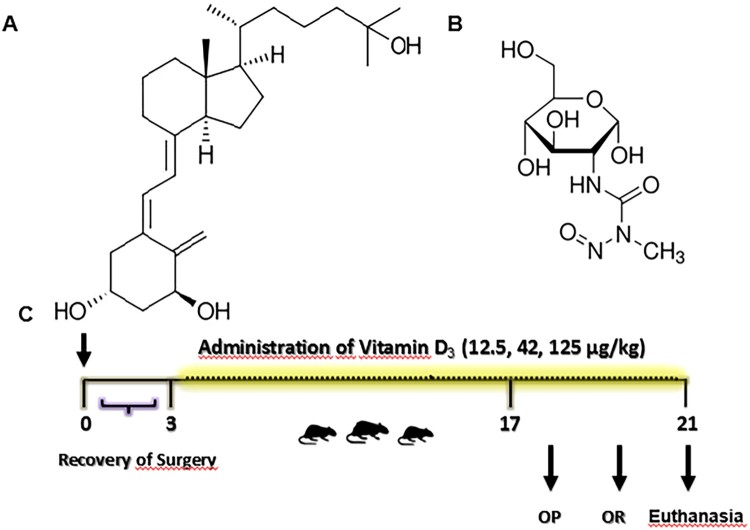
(A) Chemical structure of Vitamin D_3_ [25- Hydroxyvitamin D_3_ or (C_27_H_44_O_2_)], (B) struture of Streptozotocin [*N*-(methylnitrosocarbamoyl)-α-d-glucosamine or (C_8_H_15_N_3_O_7_)], (C) schematic representation in days of the experimental design. Day 0 indicates the day of surgery (icv- STZ infusion).

The animals were anesthetized (ketamine and Xylazine 0.5 mg/kg) intraperitoneally and positioned in the stereotaxic solution, an incision was made in midline sagittal, according to [26]. Injection was administered into the bilateral ventricle with a 28-gauge Hamilton® syringe attached to the apparatus. STZ groups received bilateral injection of ICV/STZ (3 mg/kg) dissolved in citrate buffer (pH 4.4) [27], control group received injection of ICV/citrate (Figure 1). The concentration of STZ/citrate was 5 μL/site.

### Post-operative care

All animals received dipyrone 25 mg/kg (8/8 hours/3 days).

### Experimental procedure

For assays, animals were divided into eight groups (seven animals): Vehicle, 12.5VD, 42VD, 125VD, STZ, STZ + VD12.5, STZ + VD42, STZ + VD125. For behavioral tests, animals were divided into four groups (10 animals): Vehicle, 125VD, STZ, STZ + VD125. Animals received VD_3_ by gavage (1 mL/kg) diluted in corn oil at doses: 12.5, 42 and 125 µg/kg body weight, per 21 days (10am). The doses of VD_3_ were chosen based on studies indicating neuroprotection [28,29]. Control groups received corn oil.

### Brain tissue preparation

Cerebral cortex was homogenized in 10 mM Tris–HCl, 0.1 mM EDTA, pH 7.4 [30]. After centrifuged (1500 *g*/4°C/15 min), the supernatant was stored (80°C) until use. Protein was determined (0.7 mg/mL) following the Coomassie blue method [31].

### AChE activity and in synaptosome’s AChE activity

The method [32] was modified according to [33]. The reaction mixture (2 mL/vol) contained 100 mM K+-phosphate buffer, pH 7.5 and 1 mM 5,5′dithio-bis-acid-nitrobenzoic (DTNB). The method is based on the formation of yellow anion, measured by absorbance (412 nm/2 min/25°C). The enzyme (40–50 µg/protein) was added to react with 0.8 mM acetylthiocholine iodide. Samples were run in duplicate or triplicate. Activities were expressed in µmol AcSCh/h/mg of protein.

### ROS

According to [34] adapted for cerebral cortex. S1 was incubated with 10 µL of 2′,7′-dichlorofluorescein diacetate (DCFH-DA). The ROS levels were determined by a spectrofluorimetric method. The oxidation of DCFH-DA to fluorescent dichlorofluorescein (DCF) was measured for the detection of intracellular ROS. The DCF fluorescence intensity emission was recorded (525 and 488 nm) of excitation 60 min after the addition of DCFH-DA to the medium. Results were expressed as ρmol DFC/mg protein.

### Lipid peroxidation

Thiobarbituric acid (TCA) reactive substances, such as 200 µL of samples of S1 (MDA-malondialdehyde 0.03 mM), 200 µL of 8.1% sodium dodecyl sulfate, 750 µL of acetic acid solution (2.5 M HCl, pH 3.5) and 750 µL of 0.8% TBA, were measured according to [35]. The mixtures were heated (95°C/90 min). Results were expressed as nmolMDA/mg protein.

### Protein carbonyl

By modified [36]. From 1 mL of homogenized, the protein precipitation using 0.5 mL of 10% TCA and centrifuged (1800*g*/5 min), discarding the supernatant. 0.5 mL of 10 mmol/L 2,4-dinitrophenylhydrazine (DNPH) in 2 mol/L HCl was added to protein precipitate and incubated (room temperature/30 min). During incubation, samples were mixed (15 min). After incubation, 0.5 mL of 10% TCA was added to the precipitate and centrifuged (1800*g*/5 min). Afterward, the precipitate was washed twice with 1 mL (ethanol/ethylacetate/1:1) and centrifuged to remove the free DNPH. The precipitate was dissolved in 1.5 mL of protein dissolving in the solution of 2 g sodium dodecylsulfate and 50 mg ethylenediamine tetra acetic acid in 100 mL/80 mmol/L phosphate buffer, pH 8.0 and incubated (37°C/10 min). The color intensity was measured using a spectrophotometer (370 nm against 2 mol/L HCl). Levels were calculated by the molar extinction coefficient (21 × 103 1/mol cm), the results were expressed as nmol/mg protein.

### Vitamin C

In S1, by [37]. Proteins were precipitated in a cold 10% TCA solution at a proportion of 1:1 (v/v) centrifuged (1800*g*/15 min). This supernatant was used. An 300 µL aliquot of sample in a final volume of 575 µL of solution was incubated (3 h/37°C) then 500 µL H2SO4 65% (v/v) was added to the medium. The product was determined using color reagent containing 4.5 mg/mL dinitrophenyl hydrazine (DNPH) and CuSO_4_ (0.075 mg/mL). Results were expressed as µg/vitaminC/g/tissue.

### Thiol groups

By [38], using S1 precipitated with 200 mL of 10% TCA and centrifugation. The assay was carried out in 1M phosphate buffer (pH 7.4). A standard curve using glutathione was constructed in order to calculate the SH (Spectrophotometer U-2001 Hitachi – Japan). Results were expressed as nmolSH/g.

### GSH

According to [39], 0.5 mL of supernatant and 4.5 mL of phosphate-EDTA buffer (pH 8.0) were added. The final assay mixture (2.0 mL) contained 100 µL of the diluted tissue supernatant, 1.8 mL of phosphate-EDTA buffer, and 100 µL of the OPT (O-phthalaldehyde) solution, containing 100 µg of OPT. After mixing and incubation (room temperature/15 min) fluorescence (420 nm) was determined (350 nm). Results were expressed as µmol/mL.

### GSSG

According to [40], a 0.5 mL S1 was incubated (room temperature/30 min) with 200 µL of 0.04M NEM to interact with GSH in the tissue. 4.3 mL of 0.1 M NaOH was added. A 100 µL of this mixture was taken for measurement of GSSG, using the procedure above (GSH assay), using 0.1 M NaOH as diluent rather than phosphate-EDTA. Results were expressed as µmol/mL.

### Morris water maze

According to [41], Morris water maze was placed on the surface water and milk to camouflage the platform. Animals were placed in the north, south, east and west positions. The platform was hidden 1 cm below the water (west). The latency to reach the platform was measured during 3 days and calculated as the mean of total time spent in each day. The rats remained on the platform for 30 s and when they failed to arrive in 1 min, they were forced to remain for (30 s).

## Statistical analysis

Parametric data were analyzed by one-way ANOVA followed by Krusal–Wallis test considered *p* < 0.05 as statistically significant in all experiments.

## Results and discussion

### AChE activity and AChE synaptosomes activity

**Figure 2.**
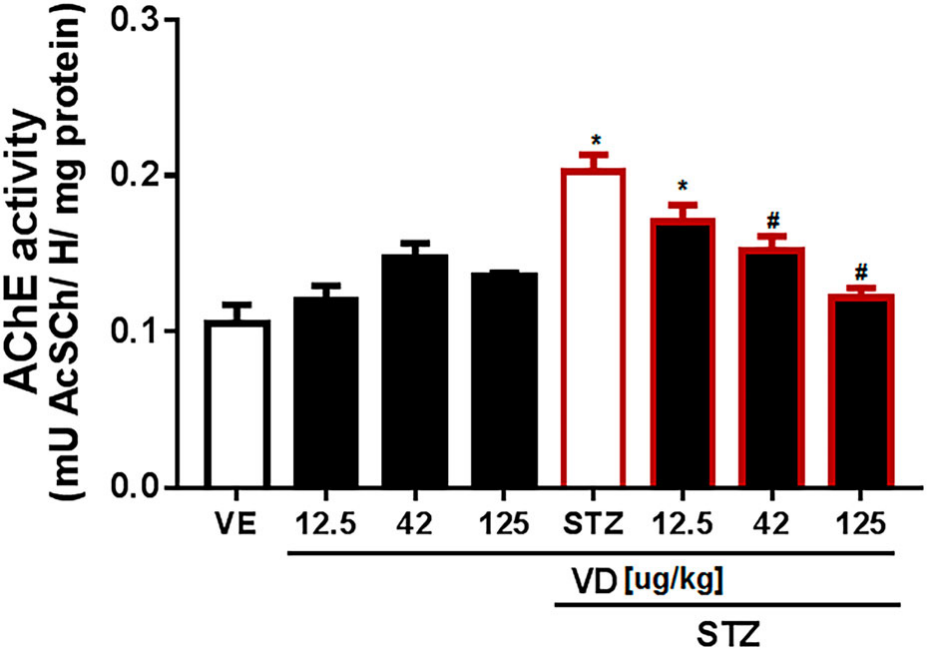
AChE activity in the cerebral cortex of rats with Sporadic Dementia of Alzheimer Type (SDAT) and treated with Vitamin D_3_ (VD) in different doses (12.5, 42 and 125 µg/kg). * indicates significant difference from the control (CTL) (*p* < 0.05). # indicates significant difference from the SDAT group (*p* < 0.05). Each column represents mean ± SEM (*n* = 7). Results are expressed as µmol AcSCh/mg protein (one-way ANOVA followed by Kruskal–Wallis).

A subdiabetogenic dose of STZ to rats causing an increase in AChE activity in the cerebral cortex (5,11,419) [*F*(7,18) = 11.33; *p* < 0.05]. However, the VD_3_ (42 and 125 µg/kg) prevents this increase (Figure 2).

**Figure 3.**
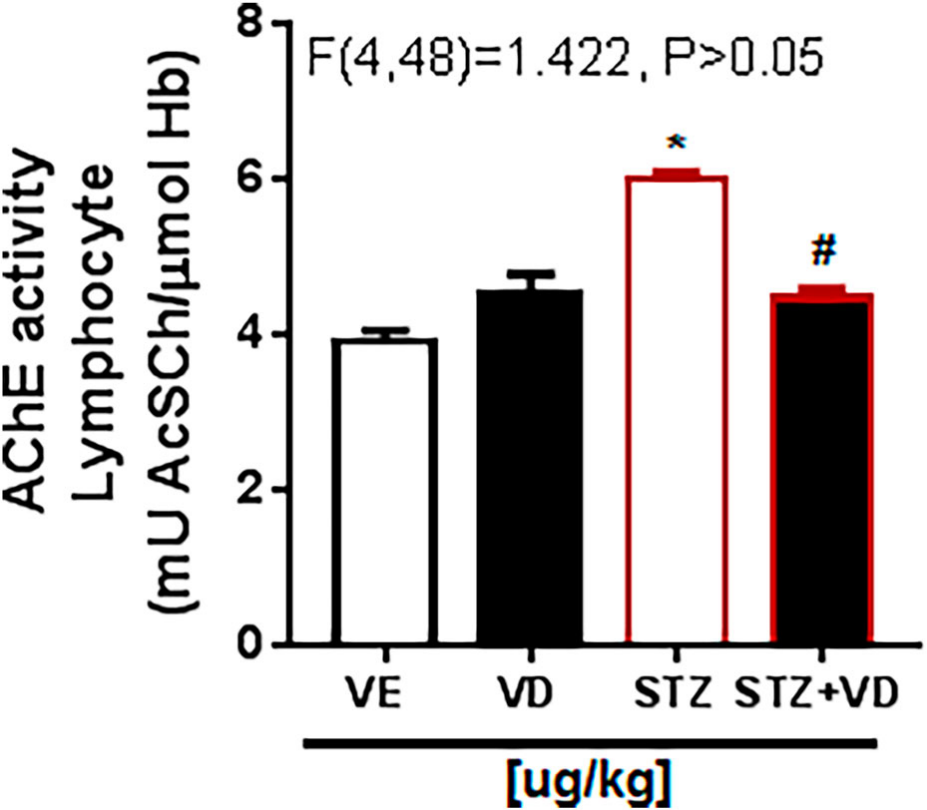
AChE activity in synaptosomes of the cerebral cortex of rats with Sporadic Dementia of Alzheimer Type (SDAT) and treated with Vitamin D_3_ (VD) in doses of 125 µg/kg. * indicates significant difference from the control (CTL) (*p* < 0.05). # indicates significant difference from SDAT group (*p* < 0.05). Each column represents mean ± SEM (*n* = 7). Results are expressed as µmol AcSCh/mg protein (one- way ANOVA followed by Kruskal–Wallis).

In the AChE activity in synaptosomes (Figure 3) there is the same increase. This result shows that the rats with ICV-STZ treated with VD_3_ (125 µg/kg) have this increase prevented [*F*(3,28) = 32.22; *p* < 0.05], this effect occurs because the VD_3_ has a potent neuro-immunomodulator, regulating the AChE activity.

The results of this study are in accordance with previous studies showing increase in AChE activity in rats with SDAT [5,6]. In addition, the impairment in insulin signaling, reduced choline acetyltransferase (ChAT) activity and increased oxidative stress induced by ICV-STZ were associated with the upregulation of AChE in the brain of the rats [5,40,41].

### ROS

According to [11,39], the subdiabetogenic dose of STZ as well as the increase in AChE activity also caused an increase in oxidative stress. Moreover, according to [7] the bilateral ICV-STZ injection results in the generation of oxidative stress and ROS in the brain of rats with SDAT.

**Figure 4.**
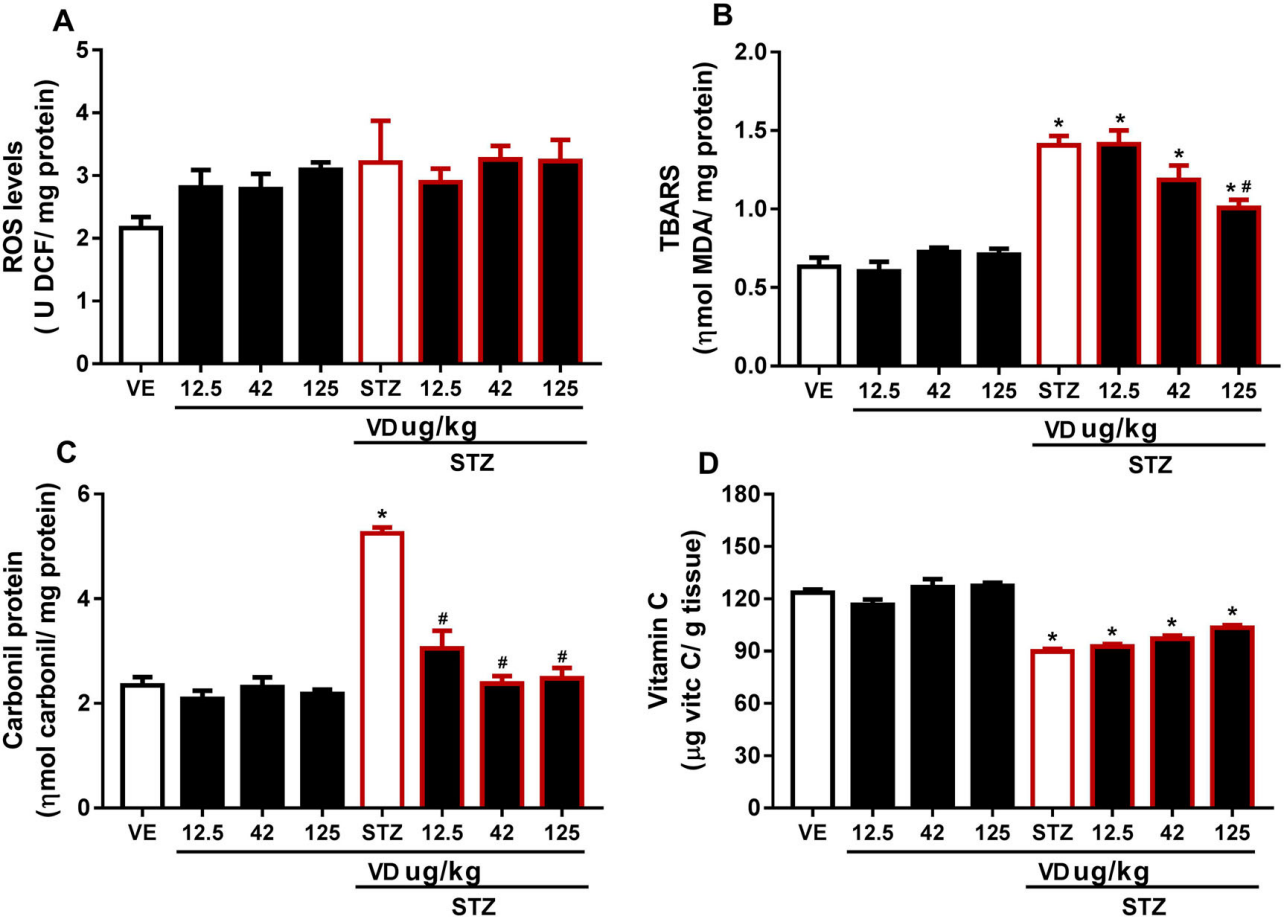
(A) ROS levels in cerebral cortex of rats with Sporadic Dementia of Alzheimer Type (SDAT) and treated with Vitamin D_3_ (VD) in different doses (12.5, 42 and 125 µg/kg). Results are expressed as ρmol DFC/mg protein (one- way ANOVA followed by Kruskal–Wallis). (B) TBARS levels in the cerebral cortex of rats with Sporadic Dementia of Alzheimer Type (SDAT) and treated with Vitamin D_3_ (VD) in different doses (12.5, 42 and 125 µg/kg). * indicates significant difference from the control (CTL) (*p* < 0.05). # indicates significant difference from the SDAT group (*p* < 0.05). Each column represents mean ± SEM (*n* = 7). Results are expressed as nmol MDA/mL (one-way ANOVA followed by Kruskal–Wallis). (C) Carbonyl protein content in the cerebral cortex of rats with Sporadic Dementia of Alzheimer Type (SDAT) and treated with Vitamin D_3_ (VD) in different doses (12.5, 42 and 125 µg/kg µg/kg). * indicates significant difference from the control (CTL) (*p* < 0.05). # indicates significant difference from the SDAT group (*p* < 0.05). Each column represents mean ± SEM (*n* = 7). Results are expressed as nmol/mg protein (one-way ANOVA followed by Kruskal–Wallis). (D) Vitamin C levels in the cerebral cortex of rats with Sporadic Dementia of Alzheimer Type (SDAT) and treated with Vitamin D_3_ (VD) in different doses (12.5, 42 and 125 µg/kg µg/kg). * indicates significant difference from the control (CTL) (*p* < 0.05). Results are expressed as µg/ vit c/g of tissue (one-way ANOVA followed by Kruskal–Wallis).

In this study, we observed no significant result in ROS levels (Figure 4(A)) [*F*(7,37) = 1.735; *p* > 0.05], but a slight increase, probably the measurement of total ROS is quite sensitive to the environment and this may have interfered with our results; however, the literature already set in postulated that ROS increased in STZ [42]. This increase probably occurs due to the fact that patients with AD are more susceptible to oxidative stress, and also because of the Ca^2+^ elevation in the brain [43]. However, studies show that the VD_3_ performs an important role in the homeostasis of Ca^2+^ [42,43], which leads us to understand this prevention causes a slight increase in ROS. In addition, the VD_3_ is able to reduce superoxide anion production [23].

### Lipid peroxidation

The LPO and protein oxidation lead to loss of membrane integrity which is an important factor in the acceleration of aging and age-related neurodegenerative disorders [44].

According to authors [11,45], this study found an increase in TBARS levels in STZ [*F*(7,20) = 24.39; *p* < 0.05]; however, VD_3_ (125 µg/kg) prevents this increase (Figure 4(B)).

The increase in TBARS levels can indicate a change in the proportion or type of fatty acids in membranes of rats with SDAT, which could provide a greater amount of substrate for LPO reactions. The VD_3_ was able to reverse this increase in TBARS levels, probably because the VD_3_ is highly lipophilic, it may accumulate in membranes to achieve the concentrations found inhibiting LPO [46].

### Carbonyl protein

The protein carbonyl content is the marker most commonly used for protein oxidation [45,47]. Accumulation of protein carbonyls has been observed in several human diseases, including AD [48]. In our study we observe elevated protein carbonyl levels in rats with STZ [*F*(7,17) = 32.69; *p* < 0.05] (Figure 4(C)), which may disclose protein damage as a consequence of ICV-STZ. The VD_3_ was able to prevent the increase in carbonyl content in all doses, probably due to its antioxidant proprieties. According to [48] oxidative stress-induced increase in protein oxidation may be corrected with VD_3_ treatment.

## Vitamin C

Vitamin C is the primary antioxidant in plasma and cells to be depleted under conditions of oxidative stress [49]. Our results showed a decrease in these levels in SDAT (Figure 4(D)) [*F*(7,16) = 31.11; *p* > 0.05]. This reduction occurs probably due to the extensive utilization of vitamin C as an antioxidant. The VD_3_ was not able to prevent the decrease; however, in the higher dose VD_3_ there was a small increase in these levels, which indicates that probably a higher dose of VD_3_ might be able to prevent.

### Thiol groups, GSH and GSSG

**Figure 5.**
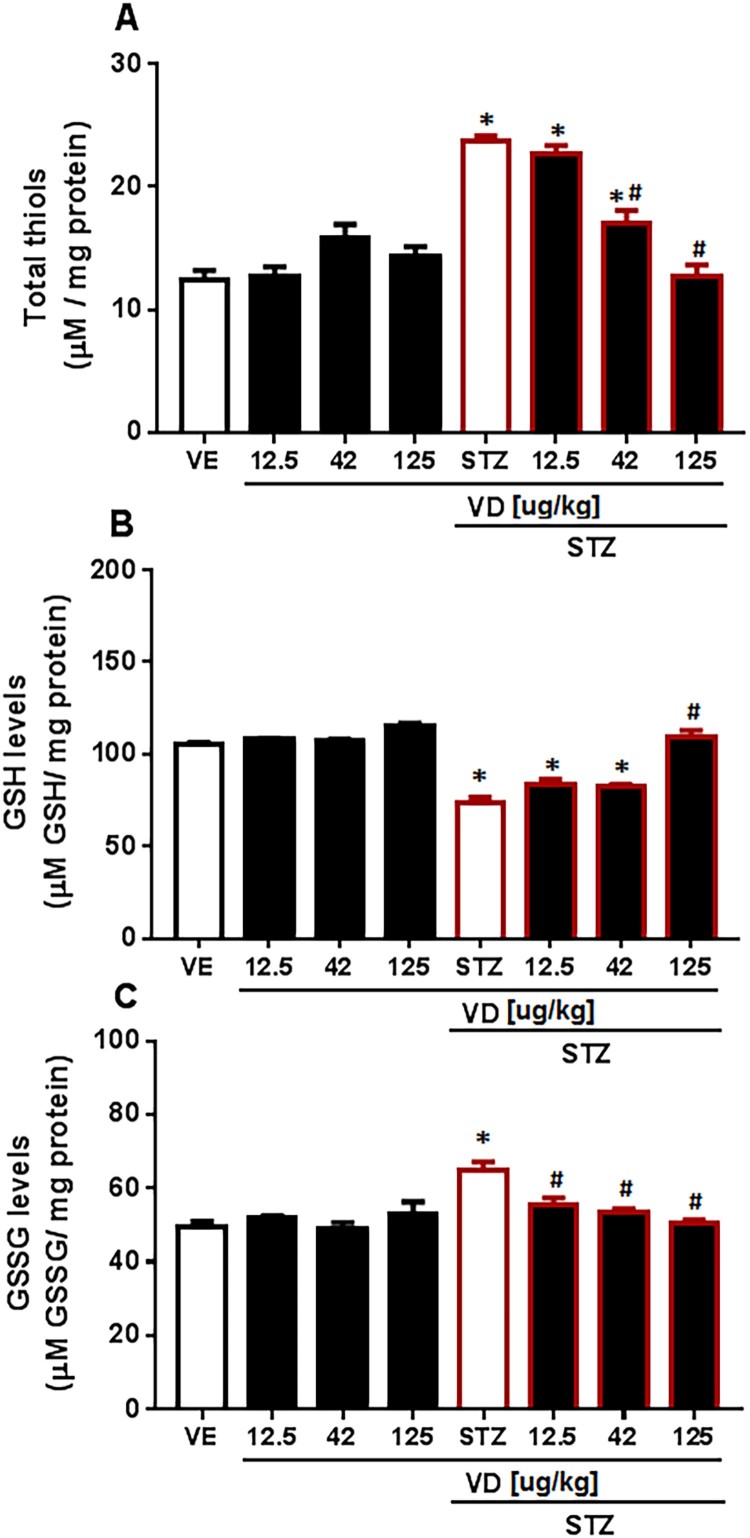
(A) Total SH content in cerebral cortex of rats with Sporadic Dementia of Alzheimer Type (SDAT) and treated with Vitamin D_3_ (VD) in different doses (12.5, 42 and 125 µg/kg µg/kg). * indicates significant difference from the control (CTL) (*p* < 0.05). # indicates significant difference from SDAT group (*p* < 0.05). Each column represents mean ± SEM (*n* = 7). Results are expressed as nmol of SH/g of tissue (one-way ANOVA followed by Kruskal–Wallis). (B) GSH levels in the cerebral cortex of rats with Sporadic Dementia of Alzheimer Type (SDAT) and treated with Vitamin D_3_ (VD) in different doses (12.5, 42 and 125 µg/kg µg/kg). * indicates significant difference from the control (CTL) (*p* < 0.05). # indicates significant difference from the SDAT group (*p* < 0.05). Each column represents mean ± SEM (*n* = 7). Results are expressed as µmol/mL (one-way ANOVA followed by Kruskal–Wallis). (C) GSSG levels in the cerebral cortex of rats with Sporadic Dementia of Alzheimer Type (SDAT) and treated with Vitamin D_3_ (VD) in different doses (12.5, 42 and 125 µg/kg µg/kg). * indicates significant difference from the control (CTL) (*p* < 0.05). # indicates significant difference from the SDAT group (*p* < 0.05). Each column represents mean ± SEM (*n* = 7). Results are expressed as µmol/mL (one-way ANOVA followed by Kruskal–Wallis).

We found a significant increase in total Thiols [*F*(7,17) = 29.88; *p* < 0.05] (Figure 5(A)). In GSH levels, a significant decrease was found in SDAT [*F*(7,29) = 0.6399; *p* < 0.05] (Figure 5(B)). The VD_3_ was able to prevent the increase in doses of 42 and 125 µg/kg. For GSSG levels an increase was found in SDAT (Figure 5(C)) [*F*(7,30) = 1.392; *p* < 0.05] and VD_3_ prevents the increase in all doses (12.5 µg/kg, 42 and 125 µg/kg). This result occurs because GSH has an important role as an antioxidant-free radical scavenger. The GSH levels make the proton donor convert the H_2_O_2_ into water and molecular oxygen by GSH–Px; in this process, GSH is oxidized to GSSG [50], which matches the results. The decrease in GSH levels occurs in many neurodegenerative diseases, including AD [51]. Studies show that the VD_3_ prevents oxidative stress in rats with AD by controlling some detoxification steps [52], probably due to it the VD_3_ reverses the decrease in GSH levels and consequently prevents the increase in GSSG levels and in total Thiols.

### Morris water maze (MWM)

***Time to Find the Platform, Time Spent in the Quadrant, Crossings in the quadrant, Time spent in the quadrant target (day test), Crossing (day test)***

The MWM task was originally designed to study the mechanisms of spatial localization in rats. Thus, spatial depends on the coordinated action of different brain regions constituting a functionally integrated neural network [53]. Besides that, it is used in models of rodents with cognitive problems, such as AD, since it evaluates the learning and memory of these animals [54,55].

**Figure 6.**
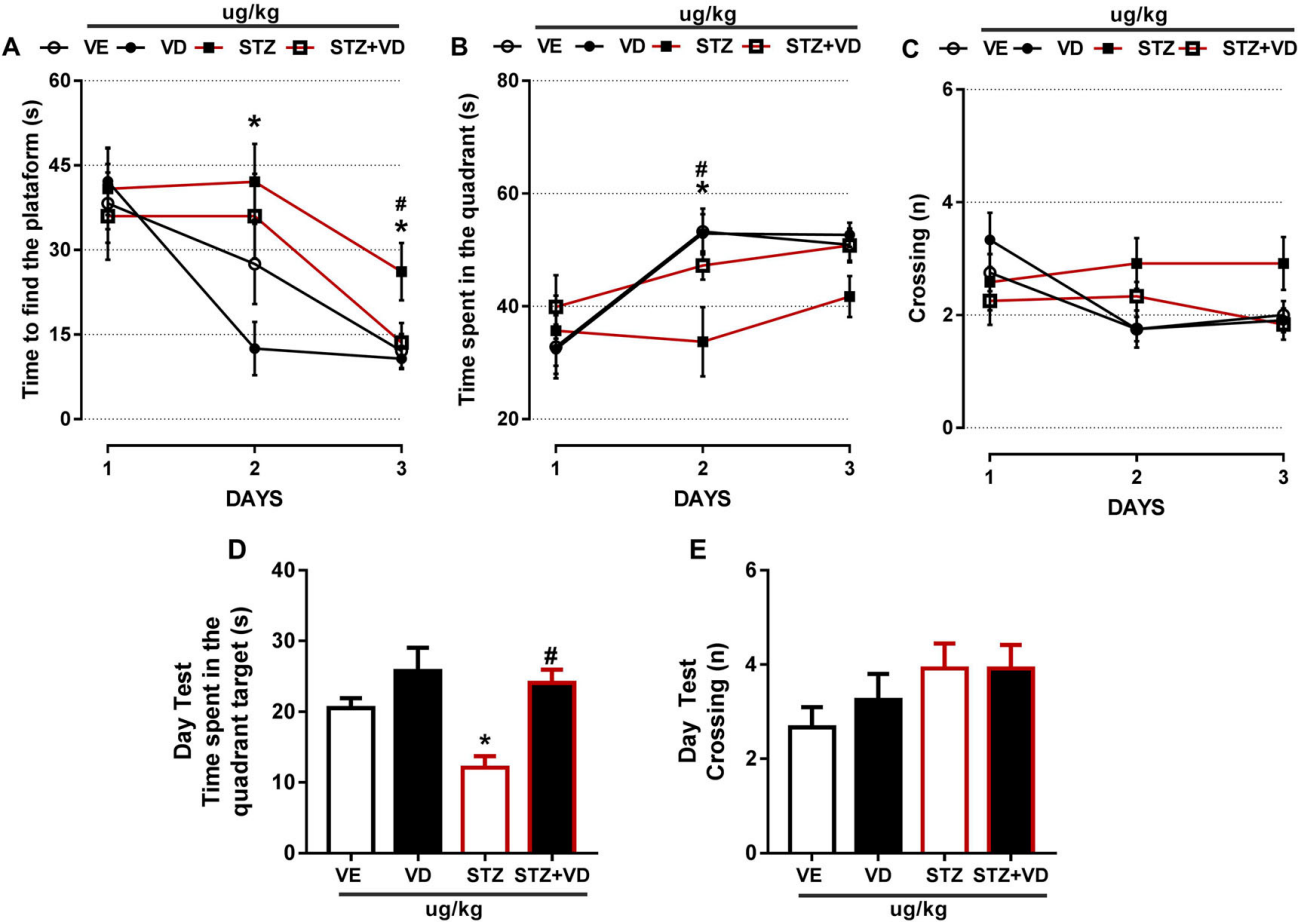
The Morris Water Maze of rats with Sporadic Dementia of Alzheimer Type (SDAT) and treated with Vitamin D_3_ (VD) in doses of 125 µg/kg. * indicates significant difference from the control (CTL) (*p* < 0.05). # indicates significant difference from the SDAT group (*p* < 0.05). Each column represents mean ± SEM (*n* = 7). Results are expressed as the time to find the platform (A), time to spent in the quadrant (B), crossings (n) (C). The Open-field tests are expressed by time spent in quadrant (s) (D) and crossings (n) (E) (One-way ANOVA followed by Kruskal–Wallis).

In relation to Time to Find the Platform, we found an increased in animals with SDAT (Figure 6(A)) in day two [*F*(3,43) = 2.935; *p* < 0.05] and day three [*F*(3,42) = 3.927; *p* < 0.05], the treatment (125 µg/kg) had beneficial effect on memory and learning in these animals only in third day.

In The Time Spent in Quadrant we found a significant difference (Figure 6(B)) only in day 2 [*F*(3,42) = 4.693; *p* < 0.05] and VD_3_ was able to prevent this increase.

The crossings (Figure 6(C)) with STZ remained the same in all days; however, the control animals had a slight decrease in day two [*F*(3,44) = 2.937; *p* > 0.05] and day three [*F*(3,43) = 2.546; *p* > 0.05]. This shows that the VD_3_ was able to prevent these crossings.

Figure 6D shows the Time Spent in The Quadrant Test, we found a significant decrease in rats with SDAT’ however, the VD_3_ was able to prevent this [*F*(4,48) = 7.831; *p* < 0.05], this result is the same with the time spent in the quadrant target (days one, two and three).

Finally, in Figure 6(E) we did not find significant difference in SDAT; however, we found a slight increase in the group that received VD_3_ [*F*(4,48) = 1.422; *p* > 0.05], this result is the same of the crossings in the previous days.

According to [54], rats that have neurodegenerative diseases and maintain their normal levels of VD_3_ have greater exploratory capacity, better learning and memory. Probably, the VD_3_ was able to reverse the deficits in learning and memory in the rats with SDAT, showing its neuroprotective effects against the neuronal damage.

## Conclusions

TBARS levels, ROS and Protein Content increase in rats with SDAT, showing an increase in oxidative stress; furthermore, the Vitamin C and GSH levels decrease, GSSG and Thiol groups increase, which indicates an altered antioxidant status in these animals. However, all enzyme activities and markers of oxidative stress were normalized with VD_3_ supplementation.

In relation to behavioral tests, these rats had deficits in learning, memory and capacity exploratory, the VD_3_ was able to prevent these deficits, showing their neuroprotective capacity.

In this way, the VD_3_ proved to be efficient in reversing oxidative stress damage and behavioral deficits in a model of SDAT, it could therefore be used as an adjuvant in the treatment of AD.
